# RasG signaling is important for optimal folate chemotaxis in *Dictyostelium*

**DOI:** 10.1186/1471-2121-15-13

**Published:** 2014-04-17

**Authors:** Alex Chattwood, Parvin Bolourani, Gerald Weeks

**Affiliations:** 1 Department of Microbiology and Immunology, University of British Columbia, 1365, Life Sciences Centre 2350, Health Sciences Mall, V6T 1Z3 Vancouver, BC, Canada

**Keywords:** Ras GTPase, Folate chemotaxis, Signalling

## Abstract

**Background:**

Signaling pathways linking receptor activation to actin reorganization and pseudopod dynamics during chemotaxis are arranged in complex networks. *Dictyostelium discoideum* has proven to be an excellent model system for studying these networks and a body of evidence has indicated that RasG and RasC, members of the Ras GTPase subfamily function as key chemotaxis regulators. However, recent evidence has been presented indicating that Ras signaling is not important for *Dictyostelium* chemotaxis. In this study, we have reexamined the role of Ras proteins in folate chemotaxis and then, having re-established the importance of Ras for this process, identified the parts of the RasG protein molecule that are involved.

**Results:**

A direct comparison of folate chemotaxis methodologies revealed that *rasG-C-* cells grown in association with a bacterial food source were capable of positive chemotaxis, only when their initial position was comparatively close to the folate source. In contrast, cells grown in axenic medium orientate randomly regardless of their distance to the micropipette. Folate chemotaxis is restored in *rasG-C-* cells by exogenous expression of protein chimeras containing either N- or C- terminal halves of the RasG protein.

**Conclusions:**

Conflicting data regarding the importance of Ras to *Dictyostelium* chemotaxis were the result of differing experimental methodologies. Both axenic and bacterially grown cells require RasG for optimal folate chemotaxis, particularly in weak gradients. In strong gradients, the requirement for RasG is relaxed, but only in bacterially grown cells. Both N- and C- terminal portions of the RasG protein are important for folate chemotaxis, suggesting that there are functionally important amino acids outside the well established switch I and switch II interaction surfaces.

## Background

Directional cell movement is important throughout the life cycle of multicellular organisms, from axon guidance during embryogenesis to wound healing in adults [1,2]. Understanding the mechanisms by which cells move will ultimately require a detailed knowledge of the components that regulate the behaviour of force-generating cytoskeletal proteins, such as actin and myosin.

The life cycle of the social amoeba, *Dictyostelium discoideum*, is dependent on directional cell movement. In the growth phase, amoebae are attracted to folate released from their bacterial prey [3]. When starved, amoebae establish a signal relay system based on cAMP that causes them to aggregate together into a multicellular structure. In both cases, two Ras proteins, RasG and RasC, have been shown to play central roles [4-7]. The localization of activated Ras to the front of the cell is one of the first responses to chemoattractant stimulation [8]. A recent study has shown that activation first occurs across the entire plasma membrane but progressively becomes more confined to the leading edge [9]. Furthermore, results from a detailed analysis of Ras activation in various *rasGEF* and *rasGAP* mutants support the idea that multiple Ras isoforms, such as RasG and RasC drive chemotaxis [9-11].

Vegetative cells lacking RasG exhibit a complex phenotype; reduced growth, reduced macropinocytosis, defects in cytokinesis, a disorganized cytoskeleton, reduced motility and reduced folate chemotaxis [5,12,13]. Removal of RasC likewise results in reduced folate chemotaxis, though the effect is somewhat milder compared to the loss of RasG [6]. However, when both RasG and RasC are removed in combination, the mutant cells exhibit identical phenotypes to those of *rasG-* cells, except that they are totally incapable of directional movement [7,14]. This suggests that RasG and RasC synergise to regulate folate chemotaxis but that RasG alone regulates cytoskeletal organization and motility [7]. Downstream of Ras activation, three pathways have been identified that are activated with similar kinetics; PI3K, TORC2 and sGC [15]. Furthermore, cells lacking RasG and/or RasC exhibit reduced activation of PI3K and TORC2 pathways, presumably because effective signaling requires direct interaction between these proteins and active Ras [8,11,16,17]. Surprisingly, however, none of these pathways are essential for chemotaxis to folate and instead serve only accessory roles in signal amplification [7]. In fact, recent evidence suggests that PI3K signaling is required only for macropinocytosis, and can actively inhibit folate chemotaxis [18,19]. A more likely candidate connecting Ras activation to the chemotaxis apparatus is PiKI, which produces PIP2. Cells lacking this protein display normal Ras activation but fail to initiate Ras-dependent responses or orientate in cAMP gradients [20]. Thus, while the genetic analysis of mutants is complicated by apparent redundancy between isoforms and an incomplete knowledge of the branching downstream pathways, there is a general consensus that Ras plays a significant role in signaling during directional sensing and migration [21]. This consensus view has, however, been challenged by a recent study that has shown *rasG-/rasC-* mutant cells are fully capable of folate chemotaxis, although different experimental conditions were used [22].

One consequence of removing RasG alone or both RasG and RasC from cells is the up-regulation of RasD [5,13], a protein whose expression is normally restricted to the developmental stages of the *Dictyostelium* life cycle [23]. RasD and RasG share 83% identity and differ by only 3 amino acids in the N-terminal 106 residues of the protein, with no variation in effector switch I or switch II domains. It is clear that this up-regulation is insufficient to prevent the observed defective vegetative cell phenotypes of *rasG- and rasG-/rasC-* mutant cells*.* However, the addition of exogenous RasD expression rescues the growth and cytokinesis defects, but not the motility and folate chemotaxis defects of these cells [5,13].

In this study we have directly compared folate chemotaxis in *rasG-/rasC-* cells, using the original experimental conditions [5,7] and the conditions used in the more recent study [22]. We have confirmed that RasG is important for optimal folate chemotaxis, and have then explored, using protein chimeras of RasG and RasD, which portions of the RasG molecule contribute to folate chemotaxis.

## Results and discussion

### Requirement of Ras signaling in folate chemotaxis in vegetative cells depends upon growth conditions

The recent observation indicating the absence of a role for Ras proteins in folate chemotaxis [22] is at odds with earlier observations that a *rasG-/rasC-* double knockout strain displayed zero chemotaxis towards a folate-filled micropipette [5,7]. In order to try to reconcile these conflicting results, we tested whether methodological differences between the two studies affect the chemotactic accuracy of the *rasG-/rasC-* cells. One important difference was that the earlier studies used cells grown axenically (the term “axenic” is used to refer to cells that obtain nutrients from liquid medium, in the absence of bacteria), while the more recent study used cells grown on bacteria. Therefore, we directly compared the folate chemotaxis indices of cells cultured axenically and on bacteria.

**Figure 1:**
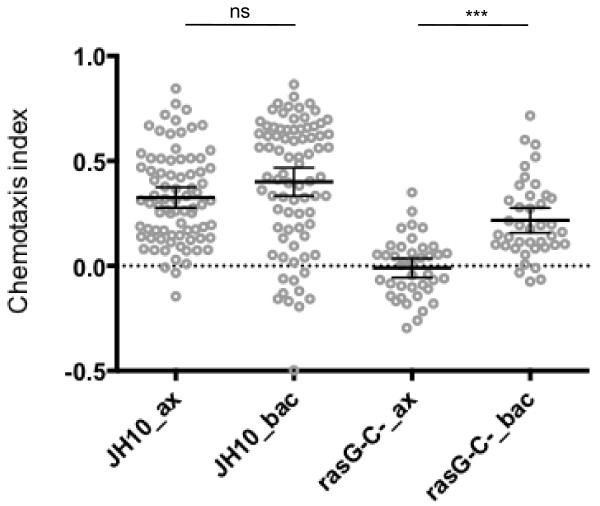
**The effect of axenic vs. bacterial growth on folate chemotaxis.** Light grey circles show the average chemotaxis index of individual control JH10 [n = 80] and *rasG-/rasC-* cells [n = 40], grown either in axenic medium or on bacteria. Error bars indicate the 95% confidence interval of the mean (middle black bar).

Our results showed that the mean chemotaxis index of *rasG-/rasC-* cells was significantly increased by the shift from axenic to bacterial growth (Figure 1, mean diff. = 0.227, 95% CI [0.092, 0.362], p = <0.0001). This result is consistent with the recent conclusion that *rasG-/rasC-* cells are capable of positive chemotaxis [22]. We noted, however, that there was still a significant reduction in folate chemotaxis between JH10 cells and *rasG*^*-*^*/rasC-* cells grown on bacteria (Figure 1, mean diff. = -0.183, 95% CI [-0.066, -0.3], p = <0.001). Furthermore, there was negligible chemotaxis by *rasG-/rasC-* cells relative to control JH10 cells, when cells were grown axenically (Figure 1, mean diff. = -0.336, 95% CI [-0.219, -0.452], p = <0.00001), supporting the earlier conclusions [5,7]. Our data, therefore, indicate that Ras signaling is important for optimal chemotaxis to folate regardless of growth conditions, but that this requirement is diminished by growth on bacteria.

### Redundancy amongst Ras proteins does not explain the enhanced folate chemotaxis of bacterially grown *rasG-/rasC-* cells

One possible explanation for the enhanced chemotaxis phenotype of *rasG-/rasC-* cells is that growth on bacteria induces cellular changes that render RasG and RasC less important during folate chemotaxis compared to other Ras proteins. The *Dictyostelium* genome encodes 11 *ras* genes. Of these, 5 (RasG, RasC, RasD, RasB, RasS) have been partially characterised, and all (except RasS) have been detected by specific antibodies during the growth phase and shown to have different mobilities on electrophoresis gels [13,24]. Extremely low transcript levels argue that the remaining 6 *ras* gene products are unlikely to be detectable by Western Blot (DictyExpress). Therefore, the levels and identities of different Ras proteins can be identified using a non-specific Pan-Ras antibody.

**Figure 2:**
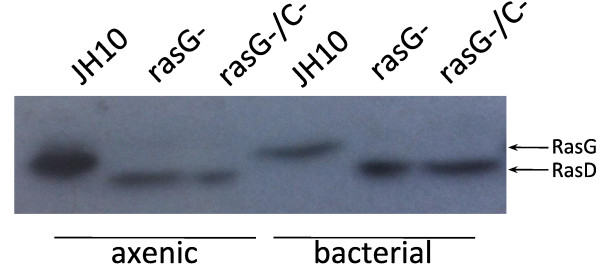
**Total Ras expression in axenic and bacterially grown cells.** Levels determined with Pan-Ras antibody. The lower M_r_ bands in the ras*G-* and ras*G-rasC-* lanes represents up-regulated RasD (arrow).

Firstly, there was no evidence of any novel Ras isoform in the bacterially grown *rasG-/rasC-* cells when compared to their axenic counterparts (Figure 2). This result suggests that any Ras-mediated improvements to folate chemotaxis would manifest as changes in the expression level of the Ras proteins already present in these cells.

It has been shown previously that RasD expression is considerably enhanced when the *rasG* gene is deleted [5,13], and this enhanced expression is also detected by the Pan-Ras antibody. RasD levels were undetectable in the JH10 cells but clearly present in both *rasG-* and *rasG-/rasC-* cells grown axenically and on bacteria (Figure 2). In addition, bacterial growth of these cells was correlated with qualitative increases in RasD levels. This increase cannot explain the improvement in folate chemotaxis of *rasG*^*-*^*/rasC*^*-*^ cells observed by a switch from axenic to bacterial growth, since exogenous RasD expression does not rescue the chemotactic defects of *rasG*^*-*^*/rasC*^*-*^ cells [5,13].

Surprisingly, there was a comparative decrease in RasG levels in JH10 cells grown on bacteria. This clearly has no impact on folate chemotaxis, since their folate chemotactic indexes are not significantly different (Figure 1), and may instead be related to the significant differences in growth rates between the bacterially and axenically grown cells.

Finally, it is also unlikely that there are increased levels of active Ras in bacterially grown *rasG*^*-*^*/rasC*^*-*^ cells, because membrane recruitment of RBD-GFP remains low in cells stimulated with folate [22]. Thus, there is no evidence that the improved chemotactic accuracy of bacterially grown *rasG*^*-*^*/rasC*^*-*^ cells involves novel Ras signaling pathways or the modulation of currently existing ones.

### Initial distance from micropipette affects folate chemotaxis of bacterially grown *rasG-/rasC-* cells

**Figure 3:**
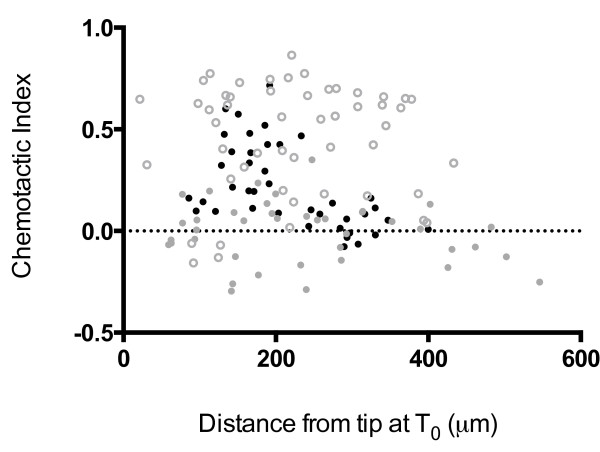
**The effect of distance on folate chemotaxis of bacterially grown *rasG-/rasC- *cells.** Chemotaxis index values plotted against distance from micropipette at T_0_. N = 40: Bacterially grown JH10 cells (open grey circles); bacterially grown ras*G-rasC-* cells (closed black circles); axenically grown ras*G-rasC-* cells (closed grey circles).

There is an additional difference in the folate chemotaxis measurements between the earlier and more recent studies. In the more recent study [22], folate chemotaxis was measured in a field of cells that appeared to be at considerably higher cell density than was used for the earlier measurements [5,7] and consequently the chemotactic index was measured only for cells close to the tip. Chemoattractant gradients decrease exponentially at increasing distances from the tip such that cells close to the tip experience high concentrations and steep gradients of folate, whereas those further away experience lower concentrations and shallower gradients [25]. This has been shown to influence chemotactic measurements [7]. We therefore controlled for the cell density of the *rasG-/rasC-* cells and looked at the effect of distance from the micropipette at T_0_ on the determination of the folate chemotaxis index. As shown in Figure 3, individual *rasG-/rasC-* cells grown on bacteria displayed positive chemotaxis below an initial distance of 200 μm (average chemotactic index of 0.33), but negligible levels of chemotaxis (average chemotactic index of 0.08) above a distance of 200 μm. Over the same distance range, JH10 cells grown on bacteria exhibited no variation (Figure 3). Thus, initial distance of the cell from the micropipette is an important factor determining whether or not bacterially grown *rasG*^*-*^*/rasC*^*-*^ cells will exhibit directional movement. In contrast, *rasG*^*-*^*/rasC*^*-*^ cells grown axenically displayed negligible chemotaxis at all distances from the micropipette (Figure 3), confirming previously published data [7]. This result reinforces the idea that Ras signaling is crucial for folate chemotaxis of axenically grown cells. Furthermore, we show that Ras signaling is not absolutely essential for the chemotaxis of bacterially grown cells, but that its role becomes increasingly important at either lower concentrations or shallower gradients of folate.

The above results indicate that there is a clear difference between the requirements of Ras for folate chemotaxis in axenically and bacterially grown cells. There have been several reports detailing morphological, metabolic and transcriptional differences between axenically and bacterially grown cells [26,27]. There are also effects on cell motility. First, bacterially grown cells move faster than axenic cells in random motility assays [28-30]. Indeed, our own measurements show that bacterially grown JH10 and *rasG-/rasC-* cells migrate at higher velocities than their axenically grown counterparts during folate chemotaxis (Additional file 1: Figure S1). Second, a recent study has shown that transfer of the background strain AX2 from axenic to bacterial growth improves chemotaxis in linear folate gradients [19]. In the same paper, PIP_3_-dependent macropinocytosis, the predominant mode of feeding in amoebae grown axenically, was identified as an inhibitor of chemotaxis. We did not observe a significant difference between the chemotactic indices of JH10 cells grown in axenic medium or on bacteria (Figure 1), possibly owing to strain-specific differences. However, there was a clear difference between folate chemotaxis of axenic and bacterially grown *rasG-/rasC-* cells (Figure 1). Whilst axenically grown *rasG*^*-*^*/rasC*^*-*^ cells exhibit reduced macropinocytosis (unpublished observations), there may still be sufficient activity to severely inhibit folate chemotaxis.

Although the effect of macropinocytosis inhibition may explain some of our observations, it does not explain why ras*G-/rasC-* cells exhibit reduced chemotaxis regardless of their growth condition. One possibility is that axenically grown cells are relatively unpolarized, and only become polarized when exposed to folate gradients (R. Insall, personal communication). Ras signaling is important for initiating and reinforcing decisions to polarize the cytoskeleton [9] and axenically grown *rasG-/rasC-* cells may be less capable of amplifying signals sufficiently to be able to orient towards the chemotactic signal. In contrast, Ras-dependent amplification of the chemotactic signal in a strong folate gradient is important for optimal chemotaxis of bacterially grown *rasG-/rasC-* cells, but not essential. Interestingly, Ras signaling is still of prime importance for the bacterially grown *rasG-/rasC-* cells in shallow folate gradients.

### Specificity of the RasG requirement for optimum chemotaxis

**Figure 4:**
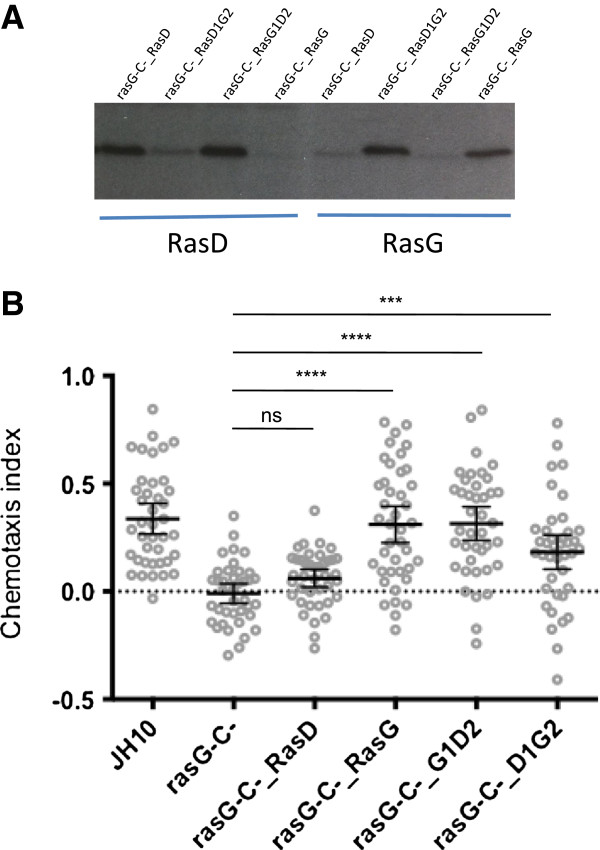
**Both N- and C- terminal halves of RasG are required for optimal folate chemotaxis of axenically grown cells. (A)** Expression of RasG/RasD protein chimeras detected by binding of RasG and RasD antibodies specific to the C-terminus. **(B)** Chemotaxis index protein chimeras and controls [n = 40]. Error bars indicate the 95% confidence interval of the mean. Statistical significance: ns = P > 0.05; *** = P < 0.001; **** = P < 0.0001. Light grey circles show average chemotaxis index of each cell. All strains were grown in axenic conditions.

We showed previously that exogenous expression of RasD was incapable of restoring folate chemotaxis in *rasG-/rasC-* cells [5,13]. To understand what makes RasG uniquely required for chemotaxis, we decided to generate chimeras of RasG and RasD that would assess the relative importance of the 3 amino acids in the N-terminal portion of the RasG protein and the differences in residues in the C-terminal half of the protein. For this, a PCR-based approach was used in which primers were designed to generate a product that would leave an overhanging end containing sequence to which a corresponding product could ligate. The product, designated RasD1G2, corresponds to the N-terminal 104 residues of RasD ligated to the C-terminal 86 residues of RasG. The product, designated RasG1D2, corresponds to the N-terminal 104 residues of RasG ligated to the C-terminal 84 residues of RasD (Note: the total sequence lengths are not the same because RasG contains two additional amino acids). Each of these products was cloned into an exogenous expression vector downstream of the *rasG* promoter and transformed into *rasG-/rasC-* cells. Intact RasD and RasG were also transformed so that there was an experimental baseline to which our chimera data could be compared. G418-resistant clones were isolated and subjected to Western blotting with specific RasD or RasG antibodies to confirm that all the proteins were expressed at similar levels (Figure 4A).

As shown previously, expression of RasG in *rasG-/rasC-* cells almost fully rescued the folate chemotaxis defect, while RasD expression had a minimal effect on chemotaxis (Figure 4B). The expression of either the RasD1G2 or RasG1D2 constructs in *rasG-/rasC-* cells significantly improves folate chemotaxis (Figure 4B). However, a statistical comparison between JH10 cells and RasD1G2 and RasG1D2 expressing cells reveals that chemotaxis is fully restored only by RasG1D2 (Figure 4B, mean diff. = 0.023, 95% CI [-0.105, 0.151], p = >0.05), and not RasD1G2 proteins (Figure 4B, mean diff. = 0.155, 95% CI [-0.027, 0.283], p = <0.05). This result suggests 1) that both the 3 altered amino acids in the N-terminal portion and the entire C-terminal portion make important contributions to RasG-mediated chemotaxis and 2) the N-terminal portion is perhaps more important for this function than the C-terminal portion. Likewise, an earlier study found that both the N-terminal and C-terminal portions of the RasC molecule were important for adenylate cyclase activation during the aggregation of starving cells towards the chemoattractant, cAMP [31]. It was suggested that both the interaction of RasC with a specific downstream effector through the N-terminal portion of the molecule and the subcellular membrane localization through its C-terminal portion were important for function. Similar conclusions may be reached regarding the specificity of RasG for folate chemotaxis in vegetative cells. However, it is important to note that the differences between N-terminal portions of RasG and RasD number only 3 amino acids. Moreover, each of these differences is conservative (Ser > Thr, Asp > Glu, Tyr > Phe), and located at positions outside of the normal switch I and switch II interaction surfaces.

Understanding the functional specificity of different Ras isoforms is a hurdle that must be overcome to develop efficacious inhibitors of Ras signaling [32,33]. Protein chimeras composed of subfamily isoforms can be used to explore portions of protein molecules that are important for specific functions. *Dictyostelium* RasD and RasG are highly homologous to human Ras proteins (64% and 67% compared to H-Ras; 65% and 70% compared to K-Ras). Therefore, an insight into what makes RasG and RasD functionally distinct from each other may open up new avenues of inquiry in higher organisms.

## Conclusion

RasG is required for optimal chemotaxis, regardless of growth condition. Increased initial distance from the chemoattractant source is correlated with reduced chemotactic accuracy, suggesting that RasG is particularly important for directional cell migration in weak gradients. In strong gradients, the requirement for RasG is relaxed, but only in bacterially grown cells. The role of RasG in folate chemotaxis is unique, and cannot be replaced by the 83% identical, RasD molecule. Both N- and C- terminal portions of the RasG protein contribute to folate chemotaxis, suggesting that there are functionally important amino acids outside the well established switch I and switch II interaction surfaces.

## Methods

### Cell culture and maintenance

For axenic growth, all strains were cultured in HL-5 medium (per litre: 15.4 g glucose, 14.3 g bactopeptone, 7.15 g yeast extract, 0.96 g Na_2_HPO_4_, 0.49 g KH_2_PO_4_) containing 50 mg/ml streptomycin (Sigma, USA) and 50 mg/ml ampicilin (Sigma, USA). Strain JH10 was further supplemented with 100 μg/ml thymidine (Sigma, USA). Strains transformed by electroporation (http://www.dictybase.org) with 20 μg exogenous plasmid were selected and maintained in HL-5 with 10 μg/ml G418 (Invitrogen, Carlsbad CA). For bacterial growth, clearing plates were prepared. In which, 5e^5^ amoebae were plated in association with 400 μl of a thick suspension of *Klebsiella aerogenes* and kept in a dark, moist box at 22°C*. C*ells were deemed ready for experimentation once the amoebae had eaten the majority of bacteria, indicated by a change of surface texture in the petri dish; from opaque and matte to transparent and glassy. Preparation of cells grown under different conditions for experimentation was as follows: Axenic cells were washed from sub-confluent tissue cultures plates (Nunc, Rochester NY) containing HL-5. Bacterially grown cells were harvested into HL-5 from clearing plates and the remaining bacteria removed by 3× 5 min centrifugation steps at 1000 rpm. Axenic and bacterially grown amoebae were henceforth treated identically in all subsequent experiments.

### Chimera constructs

All Ras proteins were cloned into vector #188, a modified version of pBS KS (Promega, WI) in which the neomycin resistance cassette and a genomic fragment containing the RasG promoter and coding sequence have been inserted. Genomic RasG was replaced with coding sequence using BglII and XhoI restriction sites, such that the RasG promoter drove the expression of the cloned fragment. RasD and RasG constructs were generated by PCR from pGEM-T-Easy (Promega, WI) templates containing RasD and RasG cDNA. These vectors also served as templates to generate the PCR fragments that were subsequently ligated to form RasD1G2 and RasG1D2 constructs, using the method and thermocycling parameters detailed in [31]. Primers used: D1-F_BglII (5′-CGC*AGATCT*ATGACAGAATATAAATTA-3′), D2-F (5′-AAAGATAGAGTACCATTGATTTTGG-3′), D2ovr-G1R (5′-CAATGGTACTCTATCTTTATCCTTAACTCTAAGAATTTGTTC-3′), D2-R_XhoI (5′-AGG*CTCGAG*TTATAAAATTAAACATTG-3′), G2-F (5′-AAGGATAGAGTACCAATGATTGTCG-3′), G2-R_XhoI (5′-CGT*CTCGAG*TTATAAAAGAGTACAAG-3′), G2ovr_D1R (5′-CATTGGTACTCTATCCTTGTCTTTAACTCTTAGAATTTGTTC-3′). Note that *italics* designate restriction sites and underlined symbolizes overhangs. Primer combinations: RasD = D1-F_BglII + D2-R_XhoI; RasG = G1-F_BglII + G2-R_XhoI; RasD1 = D1-F_BglII + G2ovr_D1R; RasD2 = D2-F + D2-R_XhoI; RasG1 = G1-F_BglII + D2ovr_G1R; RasG2 = G2-F + G2-R_BglII. Products D1 + G2 were ligated to generate RasD1G2 and products G1 + D2 were ligated to generate RasG1D2 fragments.

### Western blots

Cells harvested from axenic medium and bacterial clearing plates were resuspended to a density of 1e^7^ cells/ml in 1× HK-LB (10% glycerol, 150 mM NaCl, 10 mM Na_2_PO_4_ pH7.2, 10 mM MgCl_2_, 5 mM NaF, 1 mM Na_3_VO_4_, 1 mM EDTA, 1% Triton X-100, 0.05% SDS). Protein concentration was determined by DC Assay (BioRad, CA), 6× SDS-PAGE buffer (350 mM Tris-Cl pH6.8, 30% glycerol, 10% SDS, 0.01% bromophenol blue) was added and samples were boiled for 5mins. 20 μg protein were loaded into each lane and fractionated by SDS-PAGE. After electrophoresis, proteins were transferred to a Hybond-P membrane (Amersham) and equal loading was verified by Ponceau S (BioRad, CA) staining. Membranes were blocked with 5% non-fat milk solution, and probed with antibody. To compare Ras expression in axenic and bacterially grown cells, membranes were probed overnight at 4°C with 1:1000 Anti Pan-Ras primary antibody (CalBiochem cat# op400), followed by 1 hr room temperature binding of 1:5000 Anti mouse secondary antibody (GE Healthcare cat# NA931). To examine the expression of chimeric Ras proteins, specific RasD and RasG antibodies were used at concentrations of 1:300 and 1:500, respectively.

### Folate chemotaxis

Cells harvested from axenic medium and bacterial clearing plates were suspended in antibiotic-free HL-5, deposited onto 6 cm tissue culture plates at a density of 4e^5^ cells/cm^2^ and allowed to attach for 15 mins. Media was replaced with 20% HL-5 and an Eppendorf Femtotip micropipette filled with 25 mM folate was positioned in the same focal plane as the cells. Cell movement was captured at 30s intervals by time-lapse microscopy.

### Chemotaxis analysis

Cell tracking was performed on randomly chosen cells using the mTrackJ plugin [34] in ImageJ. Coordinate information from each cell was transformed into chemotactic metrics in Microsoft Excel and graphed in GraphPad Prism. A single chemotactic index datapoint is the cosine of the angle between a line connecting a cell to the tip at time, *n*, and a line connecting a cells position at time, *n*, to its position at *n* + 1. The mean chemotactic index was determined from the sum total of indices in each track. A track was deemed complete if the cell remained in close proximity to the pipette for four consecutive frames. A score of 1 indicates perfect chemotaxis.

## Competing interests

The authors declare that they have no competing interests.

## Authors’ contributions

AC conceived and designed experiments, acquired data, analyzed and interpreted data, drafted the manuscript. PB conceived and designed experiments, acquired data, analyzed and interpreted data, drafted the manuscript. GW conceived and designed experiments, interpreted data, drafted the manuscript. All authors read and approved the final manuscript.

## Supplementary Material

Additional file 1: Figure S1 The effect of axenic vs. bacterial growth on cell velocity. Error bars indicate the 95% confidence interval of the mean. Light grey circles show average velocity of each individual cell [n = 40]. Click here for file
