# Supplementary figures and images for: RasG signaling is important for optimal folate chemotaxis in Dictyostelium
Source: BMC Cell Biol. 2014 Apr 17;15:13. doi: 10.1186/1471-2121-15-13 (PMC4021067; doi:10.1186/1471-2121-15-13)

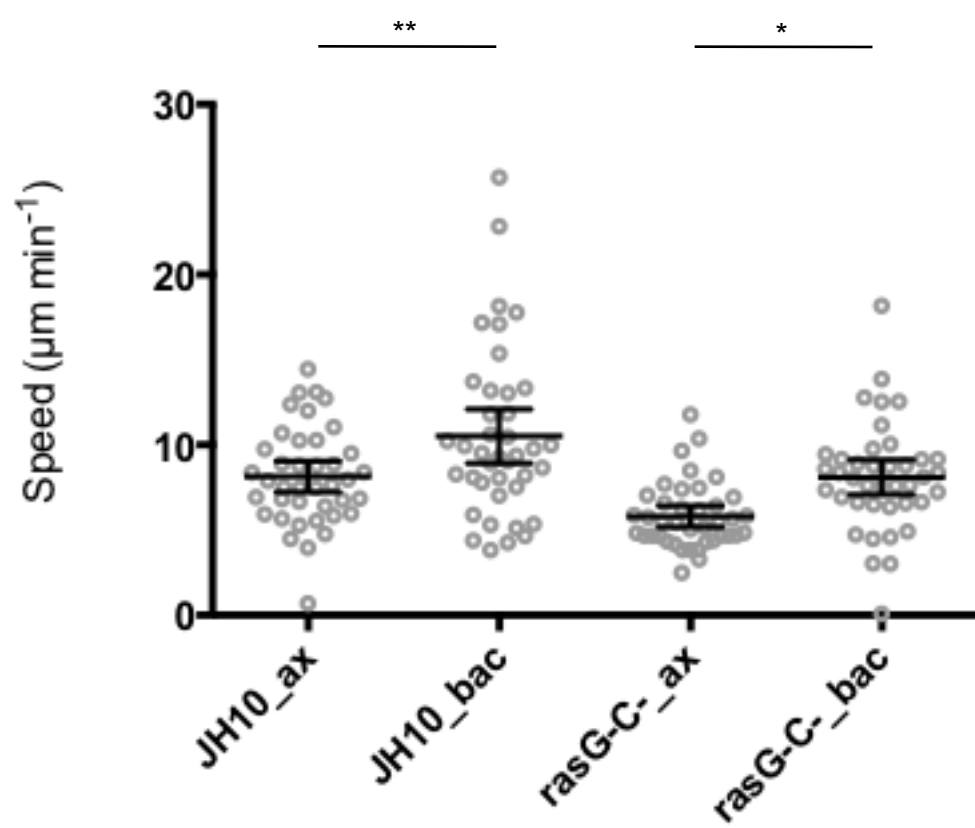

Supplement: Additional file 1: Figure S1 — The effect of axenic vs. bacterial growth on cell velocity. Error bars indicate the 95% confidence interval of the mean. Light grey circles show average velocity of each individual cell [n = 40]. [file 1471-2121-15-13-S1.pdf]
